# Pancreatic α-Cell Specific Deletion of Mouse Arx Leads to α-Cell Identity Loss

**DOI:** 10.1371/journal.pone.0066214

**Published:** 2013-06-13

**Authors:** Crystal L. Wilcox, Natalie A. Terry, Erik R. Walp, Randall A. Lee, Catherine Lee May

**Affiliations:** 1 Department of Pathology and Laboratory Medicine, Children’s Hospital of Philadelphia, Philadelphia, Pennsylvania, United States of America; 2 Department of Pediatrics, Division of Gastroenterology, Children’s Hospital of Philadelphia, Philadelphia, Pennsylvania, United States of America; 3 Department of Pathology and Laboratory Medicine, University of Pennsylvania School of Medicine, Philadelphia, Pennsylvania, United States of America; University of Bremen, Germany

## Abstract

The specification and differentiation of pancreatic endocrine cell populations (α-, β-, δ, PP- and ε-cells) is orchestrated by a combination of transcriptional regulators. In the pancreas, *Aristaless-related homeobox* gene (*Arx*) is expressed first in the endocrine progenitors and then restricted to glucagon-producing α-cells. While the functional requirement of *Arx* in early α-cell specification has been investigated, its role in maintaining α-cell identity has yet to be explored. To study this later role of *Arx*, we have generated mice in which the *Arx* gene has been ablated specifically in glucagon-producing α-cells. Lineage-tracing studies and immunostaining analysis for endocrine hormones demonstrate that ablation of *Arx* in neonatal α-cells results in an α-to-β-like conversion through an intermediate bihormonal state. Furthermore, these *Arx*-deficient converted cells express β-cell markers including *Pdx1, MafA,* and *Glut2*. Surprisingly, short-term ablation of *Arx* in adult mice does not result in a similar α-to-β-like conversion. Taken together, these findings reveal a potential temporal requirement for *Arx* in maintaining α-cell identity.

## Introduction

During development, the pancreas organizes into two distinct compartments: the exocrine acinar cells, which secrete digestive enzymes, and the hormone producing endocrine cells organized into islets of Langerhans [1]. These islets contain a core of insulin-producing β-cells with a surrounding mantle of α, δ, ε, and PP-cells, which produce the hormones glucagon, somatostatin, ghrelin, and pancreatic polypeptide, respectively [2]. Islet β- and α-cells are the two key endocrine cell populations involved in maintaining glucose homeostasis [3]. Disruption of this homeostasis through β-cell loss or dysfunction leads to diabetes mellitus, a common metabolic disorder manifested at all ages.

Given the limited supply of functioning β-cells in diabetics, one potential treatment avenue is cell-replacement therapy [4]. Considerable effort has been invested in identifying alternative β-cell sources through either directed differentiation from embryonic/induced pluripotent stem cells or reprogramming from other differentiated cell types [5]. Due to the close lineage relationship between α- and β-cells, the reprogramming potential of an α-cell to adopt a β-cell fate has been recently investigated [3]. In one study, new β-cells were generated from glucagon-producing α-cells through a glucagon^+^insulin^+^ bihormonal intermediate state after a near-total β-cell loss [6]. Moreover, an α-to-β-cell lineage conversion was observed when *Pax4*, a pro-β-cell transcription factor, was expressed in pancreatic endocrine progenitors or α-cells [7]. Similarly, forced expression of *Pdx1* in endocrine progenitors leads to an increase in β-cells and a decrease in α-cell number [8]. Although the α-cell population is mostly post-mitotic, these studies collectively illustrate that α-cell fate can be plastic and is able to be reprogrammed to adopt β-cell fate. However, the extent of this plasticity during different stages of an animal’s life is currently unknown.

One transcription factor capable of altering plasticity in endocrine cells is the *Aristaless-related homeobox* gene (*Arx*). In the mouse pancreas, *Arx* is expressed in a subset of endocrine progenitors and then restricted to glucagon-producing α-cells where it is expressed throughout the life of the animal [9], [10]. When misexpressed in the developing pancreas, *Arx* is sufficient to force endocrine progenitors or β-cells to adopt an α-cell fate [11]. These results demonstrate that *Arx* is sufficient for β-to-α-cell reprogramming during development.

Although much is known regarding factors necessary and sufficient for endocrine development, the factors required to maintain the identity of mature α-cells during different stages are less clear. Mice with *Arx* null mutations in the germ-line, pancreatic progenitors, or endocrine progenitors all display a complete loss of α-cells with a concurrent increase in β- and δ-cells in the pancreas [9], [10], [12]. Moreover, α-cell loss has been reported in patients with null mutations in *ARX* [13]. However, none of the existing mouse models are suitable for determining the function of *Arx* in maintaining (as opposed to establishing) mature α-cell identity. Further, lineage-tracing experiments have not yet been performed to determine if loss of *Arx* leads directly to an α-to-β-cell conversion.

Here we show that *Arx* is required for α-cell lineage maintenance in the neonatal pancreas, but not in the adult pancreas. During the neonatal period, ablation of *Arx* results in loss of glucagon expression and activation of insulin and β-cell markers through an insulin^+^glucagon^+^ bihormonal intermediate. In contrast, short-term *Arx* ablation in the adult pancreas does not result in either a loss of glucagon expression or an activation of β-cell marker expression. These data suggest that *Arx* may act in a stage- and context-specific manner in maintaining α-cell identity and reveal potential differential plasticity between fetal and adult α-cells. When taken together, these findings have important implications for the potential use of α-cells for the purpose of β-cell replacement therapy.

## Materials and Methods

### Ethics Statement

The Children’s Hospital of Philadelphia’s Institutional Animal Care and Use Committee (IACUC) approved all animal experiments under the protocol number 2011-10-756. CLM monitored all animal studies.

### Animals and Breeding Strategy

The derivation of the *Arx^L^/_Y_* and *Glucagon-Cre* transgenic lines has previously been described [14], [15], [16]. To generate *Arx^L^/_Y_;Glucagon-Cre* mice, *Arx^L^/_+_;Glucagon-Cre* and *Arx^L^/_Y_* mice were mated on a BL6 background. Male and female *Arx^L^/_Y_* or *Arx^L^/_L;_Glucagon-Cre* mice were phenotypically indistinguishable in terms of their islet morphology, size, body size and weight. All mutants used in our analysis were compared to their sex-matched controls. *Arx^+^/_Y_;Glucagon-Cre*, *Arx^+^/_Y_*, *Arx^L^/_+_* and *Arx^L^/_+_;Glucagon-Cre* mice were used for controls with no observable phenotypic differences in the islets between any of them. The reporter *Rosa26^YFP^/_YFP_* was mated into this line in either heterozygosity or homozygosity for lineage tracing studies, which yielded the same result in all experiments [17]. The generation of *pCAGG-CreER* animals has been previously described [18]. *Arx^L^/_Y_* or *Arx^L^/_L_;pCAGG-CreER* animals were generated by crossing *Arx^L^/_+_;pCAGG-CreER* females to *Arx^L^/_Y_* males. Male and female mutants were phenotypically indistinguishable in the endocrine pancreas and both were used in this study.

### Immunohistochemistry and Histology

All dissections were performed in cold 1×PBS and tail or toe snips collected for genotyping. Tissues were fixed in cold 4% paraformaldehyde overnight at 4°C, embedded in paraffin, and 8 µm sections collected. Antigen retrieval was performed in 10 mmol citric acid buffer (pH 6.0) and endogenous peroxidase, avidin D, and biotin activity blocked with 3% H_2_O_2_ (Sigma) and Avidin/Biotin Blocking Kit (Vector), respectively. Endogenous protein was blocked with CAS-Block reagent (Invitrogen). Slides were incubated in primary antibody overnight at 4°C. Primary antibodies used were: Insulin (MS 1∶400, Thermo Scientific and GP 1∶1000, Abcam), Glucagon (1∶3000, Millipore), Somatostatin (1∶200, Invitrogen), PP (1∶200, Invitrogen), Arx (1∶250, gift from Dr. Kanako Miyabayashi at Kyushu University), GFP (1∶250, Abcam), Ghrelin (1∶200, Santa Cruz), Pdx1 (1∶200, Santa Cruz), MafA (1∶1000, Bethyl), Glut2 (1∶1000, Millipore), and Chromogranin A (1∶3000, DiaSornin). After rinsing in PBS, appropriate secondary antibodies were added for two hours at room temperature. Immunohistochemical detection was performed with the VECTASTAIN ABC kit (Vector Laboratories) and diaminobenzidine tetrahydrochloride (DAB) as the substrate. Immunofluorescence utilized secondary antibodies conjugated to Cy3, Cy2 or Cy5. All images were obtained using a Leica DM6000B microscope.

### Real-Time PCR Analysis

Total RNA was extracted in TRIZOL (Invitrogen) using the protocol provided with reagent. Oligo-dT, Superscript II, and additional required reagents were used to synthesize cDNA (Invitrogen). PCR reactions were performed using Brilliant SYBR Green PCR Master Mix (Agilent) in the Stratagene Mx3005P real-time PCR machine. All PCR reactions were performed in duplicate for each sample with at least 3 animals per group analyzed with reference dye normalization. Primer sequences are available upon request.

### Hormone Cell Quantification

Hormone-positive cells from pancreatic sections were counted, averaged, and normalized to either total pancreatic area or total endocrine cell number. Three separate regions of each pancreas were used for quantification in both control and mutant mice. At least three animals for each group were used for quantification in all analyses. To determine hormone cell mass, hormone-positive area as well as pancreatic area was measured using the Aperio Image Analysis System. These areas as well as weight of the pancreas was used to determine hormone cell mass. For specific hormone cell number, hormone positive cells were counted and normalized to total endocrine cell number, which was determined by combining counts for all endocrine hormones (insulin, glucagon, somatostatin, and PP). Over 10,000 total endocrine cells were counted for each analysis consisting of over 5,000 insulin^+^, over 1,000 glucagon^+^ and somatostatin^+^, and over 500 PP^+^ cells.

### Islet Isolation

For P21 and adult RNA analysis, islet isolation was performed by injecting 5 mL Collagenase P (Roche) in HBSS with 0.02% BSA (Sigma) into the clamped pancreatic duct to inflate the pancreas. Once inflated and removed, pancreatic tissue was incubated in 15 mL CollagenaseP/HBSS at 37°C at 50 rpm for 16 minutes to digest exocrine tissue. After spin down and rinse, islets were isolated from remaining exocrine tissue in HBSS. Upon isolation, islets were placed in TRIZOL for RNA extraction.

### Tamoxifen Induction

Two-month-old male and female mice, matched with littermate controls, were injected intraperitoneally (IP) with 50 µg/g body weight of 10 mg/ml tamoxifen (Sigma) solution, which consisted of 10% ethanol and 90% sunflower seed oil (Sigma). Injections were performed for three consecutive days followed by a two-week chase. After the chase period, pancreatic tissue was removed and processed for either immunostaining or RNA analysis.

### Statistical Analysis

All values are presented as average ± standard error of the mean. Significance was determined using a two-tailed Student’s t-test. *p*-values less than or equal to 0.05 were considered significant.

## Results

### *Arx* Removal in Neonatal Glucagon-producing Cells

**Figure 1:**
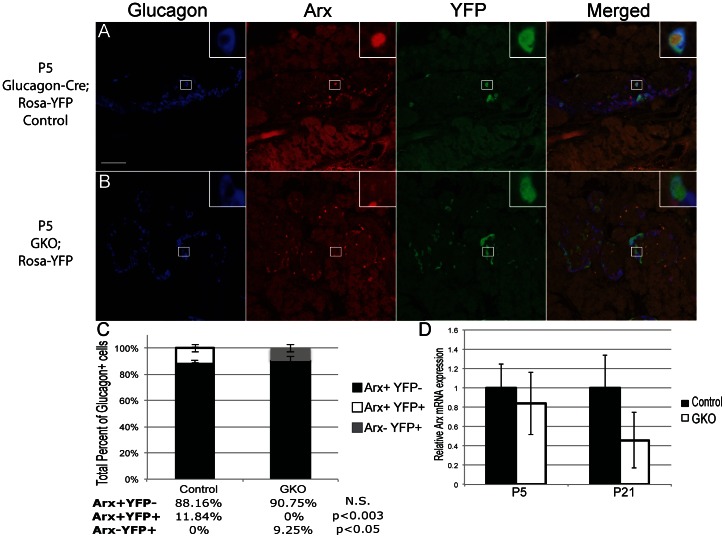
Arx is specifically ablated in YFP^+^ α-cells of GKO;Rosa-YFP mice. P5 pancreatic sections were stained for glucagon (blue), Arx (red), and YFP (green). (**A**): Arx is expressed in all glucagon^+^ cells in control;Rosa-YFP pancreata. A subset of glucagon^+^Arx^+^ cells is YFP^+^. (**B**): In GKO;Rosa-YFP animals, there is a subset of glucagon^+^ cells that express YFP. These YFP^+^ cells have lost Arx expression. Scale bar represents 25 µm. (**C**): Quantitative analysis of Arx and YFP expressing cells within glucagon^+^ population in P5 animals. Over 500 total glucagon^+^ cells were counted with three mice per group used. Error bars represent standard error of the mean with *p-value* indicated. N.S: not significant. (**D**): Quantitative PCR analysis for *Arx* mRNA in total pancreata at P5 and islets from P21 control and GKO animals. Control mRNA level was set at one fold ± standard error of the mean. Male and female control and GKO animals (n≥3) were sex-matched for all analyses.

*Arx* is expressed in endocrine progenitors, α-cell precursors, and mature glucagon-producing cells of the pancreas [9], [10]. To investigate its role in the neonatal α-cell, we generated mice with *Arx* ablation in glucagon^+^ cells (*Arx* ^L^/_Y_ or *Arx* ^L^/_L_;*Glucagon-Cre*; referred to as GKO hereon). First, a *Rosa-YFP* reporter was used to assess the Cre-mediated recombination efficiency in our GKO model. In P5 control mice (including Arx^+^/_Y_;*Glucagon-Cre*, *Rosa-YFP* and *Arx* ^L^/_+_ or ^+^/_+_; *Glucagon-Cre*;*Rosa-YFP*), Arx expression was found in all glucagon^+^ cells; however, only 13% of glucagon^+^ cells co-expressed Arx and YFP (Fig. 1A, C; white bar in control). All male and female control animals utilized in these experiments were phenotypically identical according to their islet morphology and were compared to their sex-matched GKO animals. These observations indicate a low Cre-mediated recombination rate, which is in agreement with what others have previously reported using this *Glucagon-Cre* transgenic mouse [19]. In GKO*;Rosa-YFP* mutant mice, Arx protein was removed in all glucagon^+^YFP^+^ cells, which equated to about 12% of glucagon^+^ cells (Fig. 1B, C; grey bar in GKO). This result suggests that YFP expression faithfully marks cells that have undergone *Arx* ablation. The low Cre-mediated recombination frequency was also observed in P21 animals (Fig. S1 and data not shown). Real-time PCR analysis from P5 and P21 animals further showed *Arx* mRNA levels were decreased by 20% and 50% in GKO animals, respectively, although not significantly (Fig. 1D). In addition, in P5 control animals, while 90% of YFP^+^ cells co-expressed glucagon, approximately 10% of the YFP^+^ cells were positive for insulin staining due to some leakiness of the Cre (see below and data not shown).

### *Arx* Ablation in the GKO Mice Results in an Emergence of Glucagon^+^ Insulin^+^ Co-Expressing Cells

**Figure 2:**
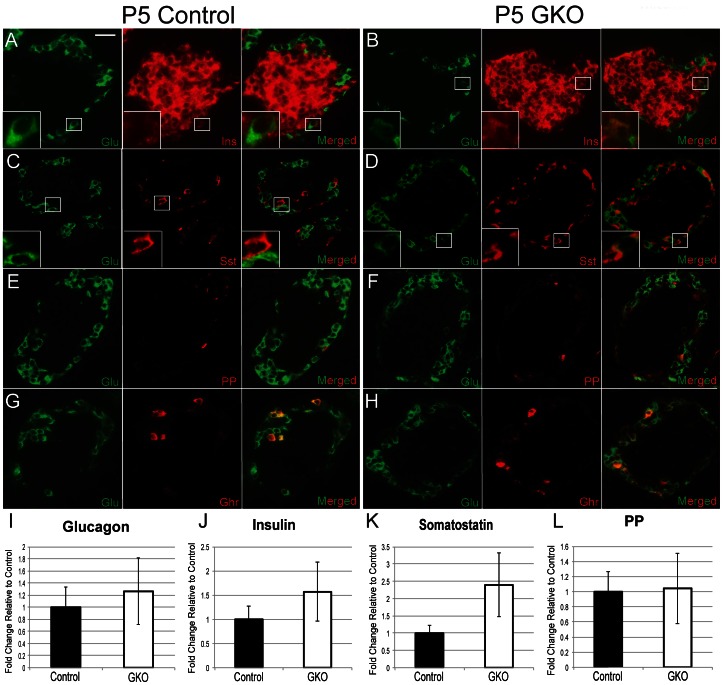
Loss of Arx in glucagon^+^ cells results in the appearance of a glucagon^+^insulin^+^ population. **(A–H):** P5 control and GKO pancreata were stained for glucagon (green), insulin (red; A, B), somatostatin (Sst; red; C, D), PP (red; E, F), and Ghrelin (red; G, H). Glucagon^+^insulin^+^ cells are the only bihormonal population unique to GKO animals (B, D, F). Glucagon/ghrelin coexpressing cells are both found in control and GKO animals (G, H). Male and female control and GKO animals (n≥3) were sex-matched for all analyses. Scale bar denotes 25 µm. (**I–L**): Quantitative PCR analysis examining glucagon (I), insulin (J), somatostatin (K), and PP (L) gene expression in P5 control and GKO animals. Control mRNA level was set at one fold ± standard error of the mean. For all GKO and control groups, at least 3 biologic replicates were performed.

To determine whether loss of *Arx* in the α-cells of GKO mice may have resulted in a change of cell fate in a small subset of cells, we performed double immunostaining for glucagon and other endocrine hormones. Given that only 12% of the glucagon-producing cells have lost Arx expression, we did not expect to observe any significant changes in the number of glucagon cells or the localization of these cells in P5 GKO mice. Indeed, immunostaining analyses confirmed this anticipated result (Fig. 2A–H). Real-time PCR analysis also revealed no significant changes in the mRNA levels of *glucagon* transcript between control and GKO mice (Fig. 2I). Although hormone cell numbers were not significantly altered, close examination revealed a small population of glucagon^+^insulin^+^ bihormonal cells in the pancreas of GKO mice (Fig. 2B). These bihormonal cells were only found in the GKO mice. There was no overlap or significant differences in the expression of glucagon with somatostatin or PP between P5 GKO and control mice (Fig. 2C–F). Endocrine cells expressing glucagon and ghrelin have been reported in the developing and neonatal pancreas [20], [21], [22]. The number and location of these glucagon^+^ghrelin^+^ cells were comparable between P5 control and GKO mice (Fig. 2G–H). Real-time PCR analysis did not reveal any significant changes in hormone expression between P5 control and GKO animals (Fig. 2I–L). Together, this data indicates that loss of Arx in glucagon^+^ cells results in misexpression of the β-cell hormone insulin in glucagon^+^ α-cells.

To determine the fate of this bihormonal population we evaluated glucagon, insulin, somatostatin, PP, and ghrelin expression in the pancreata of P21 control and GKO mice by immunostaining. Again, there was no significant change in the cell mass or distribution associated with these endocrine populations (Fig. S2A–B). Interestingly, while a few glucagon^+^insulin^+^ cells could still be found in the P21 GKO mice, the frequency of this bihormonal population was dramatically reduced by this age relative to P5. Instead, many of the remaining bihormonal cells in P21 GKO mice have reduced glucagon staining in cells readily expressing insulin (Fig. S2A–B). These cells are likely in the later stage of their α-to-β-like cell fate conversion. These observations suggest that upon ablation of *Arx*, insulin expression is activated in the glucagon-producing α-cell, which then gradually loses glucagon expression. Finally, we evaluated mRNA levels for β-cell (*Pdx1* and *Nkx6.1*) and α-cell (*MafB* and *Brn4*) markers in the islets isolated from P21 control and GKO mice. From the real-time PCR analysis, we observed a significant upregulation of *Pdx1* mRNA and an upward trend of *Nkx6.1* levels (Fig. S2J). Conversely, we detected a significant reduction in *Brn4* with a small downward trend in *MafB* expression (Fig. S2J). While the significant changes in the mRNA levels of *Pdx1* and *Brn4* data is surprising in the context of the small changes in hormone expression, this result could be due to direct *Arx* regulation. *Arx* could potentially directly repress *Pdx1* and activate *Brn4,* which would result in a drastic increase of *Pdx1* (and resulting decrease of *Brn4*) upon *Arx* ablation. When taken together, these data suggest that glucagon-producing cells require *Arx* to maintain α-cell identity and repress β-cell markers during neonatal life.

### *Arx*-deficient Cells Fail to Maintain α-cell Identity

**Figure 3:**
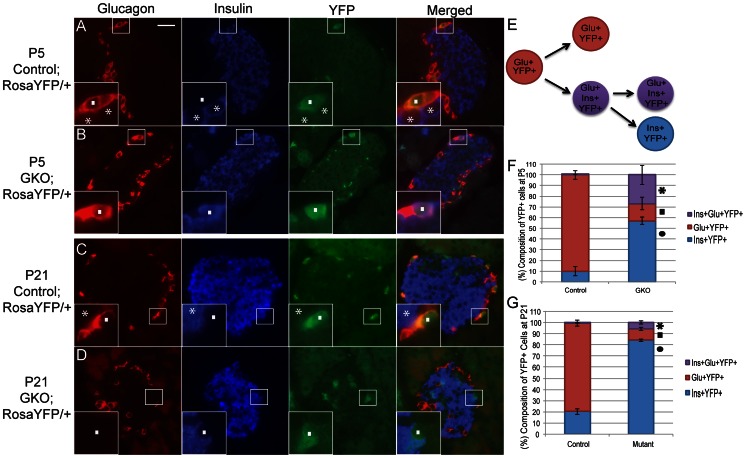
Lineage tracing studies demonstrate that Arx ablated α-cells become glucagon^+^insulin^+^ at P5 then insulin expressing at P21. (**A–D**): Triple immunostaining for glucagon (red), insulin (blue), and YFP (green) in control;Rosa-YFP and GKO;Rosa-YFP pancreata at P5 and P21. YFP^+^ cells in P5 or P21 control;Rosa-YFP animals are positive for glucagon (A and C;^▪^). “*” denote insulin cells that are negative for glucagon or YFP expression (A and C; *) Glucagon^+^insulin^+^YFP^+^ cells are found in P5 GKO animals (B; ^▪^), but rarely in controls (A). YFP^+^ cells are positive for glucagon in control P21 pancreata (C; ^▪^) but insulin^+^ in P21 GKO pancreata (D; ^▪^). (**E**): Schematic outlining cell populations resulting from lineage-tracing and immunostaining analysis. (**F, G**): Quantification of hormone expression in YFP^+^ cells at P5 (F) and P21 (G). At P5 and P21, over a total of 10,000 cells were counted from 3–5 animals per group. Out of the 10,000 cells counted, approximately 1,000 cells were YFP^+^. Each category was calculated and presented as a percentage of total YFP^+^ cells per animal and then averaged. Error bars are denoted as standard error of the mean with significance (p≤0.05) between each color denoted with “*”, “^▪^”, and “•”. Male and female GKO mice (n≥3) were used for all analysis and compared to their sex-matched controls. Scale bar represents 25 µm.

To directly determine the origin of the glucagon^+^insulin^+^ cells seen in P5 GKO mice, lineage-tracing studies were performed in P5 GKO mice. Triple-immunostaining for glucagon, insulin, and YFP were performed in the pancreas of control;*Rosa-YFP* and GKO;*Rosa-YFP* mice. YFP expression was detected in only a subset of glucagon-producing cells at P5 (Fig. 3A–B), due to the low frequency of the Cre-mediated recombination in the *Glucagon-Cre* transgenic mice (Fig. 1). The majority of YFP^+^ cells in the P5 control mice expressed glucagon (Fig. 3A, E and F) though a very small number of insulin-producing cells positive for YFP expression were found (blue), demonstrating relatively high, though not 100%, fidelity of the Cre-mediated recombination (Fig. 3E and F). In P5 GKO mice, we noticed an emergence of glucagon^+^insulin^+^YFP^+^ (purple) cells and an increase in the number of insulin^+^YFP^+^ (blue) cells while the number of glucagon^+^YFP^+^ (red) cells was reduced compared to controls (Fig. 3A–B, E and F).

To follow up with our previous observations that the glucagon^+^insulin^+^ cell number has dramatically reduced by P21, we evaluated the pancreata of control;*Rosa-YFP* and GKO;*Rosa-YFP* mice at P21 for glucagon, insulin, and YFP expression. Interestingly, corresponding to the previously described disappearance of bihormonal cells by P21 (Fig. S2A–B), the majority of YFP^+^ cells in GKO;*Rosa-YFP* pancreata at this stage were insulin^+^ (blue) with only a small percentage of YFP^+^ cells expressing both insulin and glucagon (purple) or glucagon alone (red) (Fig. 3C–D and G). These data demonstrate that *Arx* loss in glucagon-producing α-cells leads to a failure in maintaining α-cell identity and a conversion to a β-cell-like fate. Taken together, these lineage-tracing data indicate that the loss of neonatal *Arx* in glucagon-producing cells results in a cell fate conversion from a glucagon^+^ α-cell into an insulin^+^ β-cell-like fate through a bihormonal intermediate.

### Markers Associated with Mature β-cells are Activated in *Arx-*deficient YFP^+^ Cells

**Figure 4:**
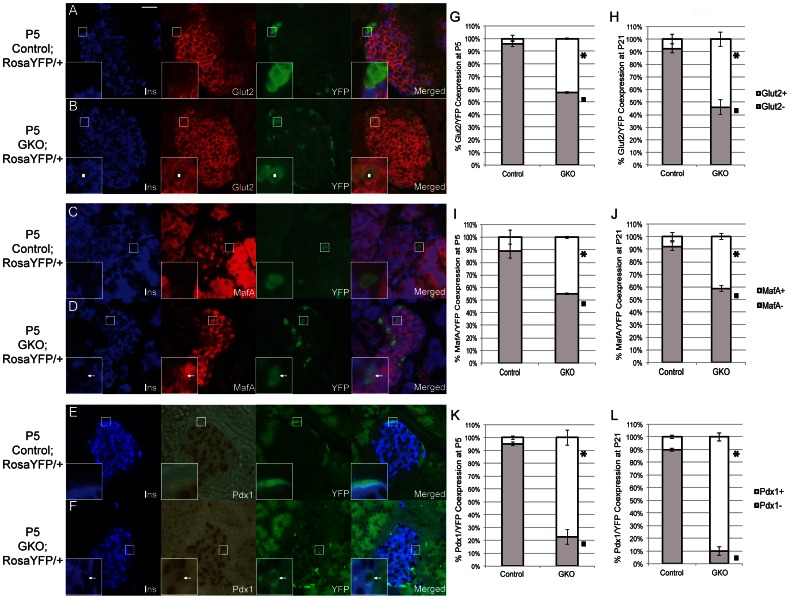
YFP^+^ cells in GKO animals express markers of mature β-cells at P5 and P21. (**A–F**): Control;Rosa-YFP and GKO;Rosa-YFP P5 pancreata were stained for insulin (blue), YFP (green), Glut2 (A,B,red), MafA (C,D,red), Pdx1 (E,F,brown). YFP^+^ cells in GKO animals are insulin^+^Glut2^+^ (B;^▪^ ), insulin^+^MafA^+^ (D; ←) and insulin^+^Pdx1^+^ (**F; ←**). In control animals, the majority of YFP^+^ do not express β-cell markers (A,C,E). The YFP^+^ cells seen in exocrine tissue (panels E and F) is background due to the combined IHC, IF staining method used and not true signal. (**G–L**): Quantification of percentage of YFP^+^ cells that express or do not express Glut2 at P5 (G) and P21 (H), MafA at P5 (I) and P21 (J), and Pdx1 at P5 (K) and P21 (L). Over 200 YFP^+^ cells were counted for each stage with 3–5 animals per group. Error bars represent standard error of the mean with significance between each cell population (p≤0.05) denoted as “*” and “^▪^”. Male and female GKO mice (n≥3) were used for all analysis and compared to their sex-matched controls. Scale bar represents 25 µm.

To further examine how closely these newly converted β-like-cells were to true β-cells, expression of several known β-cell markers including Glut2, MafA, and Pdx1 were examined in control;*Rosa-YFP* and GKO;*Rosa-YFP* mice at P5 and P21. In P5 GKO;*Rosa-YFP* animals, there was a significant increase in the number of YFP^+^ cells coexpressing Glut2, MafA, or Pdx1 (Fig. 4B, D, F, G, I, K). Similar increases were also seen in P21 mice with a further increase in the number of YFP^+^ cells expressing Glut2 and Pdx1 in the pancreata of the GKO;*Rosa-YFP* mice (Fig. 4H, J, L, Fig. S3). As expected, due to the leakiness of the *Glucagon-Cre* transgene, we did find a small, but not significant, percentage of YFP^+^ cells that coexpressed Glut2, MafA, or Pdx1 in P5 or P21 control;*Rosa-YFP* mice (Fig. 4A, C, E, G, I, K). Taken together, these data demonstrate that a subset of the converted cells in the GKO;*Rosa-YFP* mice activate β-cell markers as well as insulin expression in the absence of *Arx.*

### Short-term Ablation of Arx in Adult α-cells does not Lead to Loss of α-cell Identity

**Figure 5:**
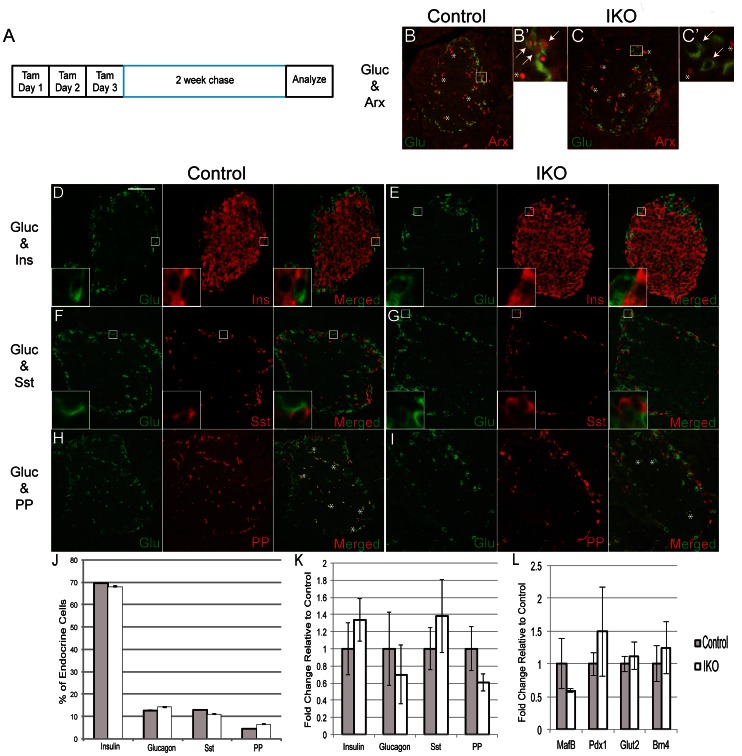
Short-term complete ablation of Arx in adult mice does not result in a loss of α-cells or changes in endocrine cell populations. (A): Diagram outlining experimental design. (**B–C**): Arx (red) is expressed in glucagon (green) cells in control (B,B’; arrows) but lost in IKO (C,C’;arrows) animals. Asterisks (*) mark autoflourescent blood cells and are non-specific staining. (**D–I**): Control and IKO pancreata were stained for glucagon (green), insulin (red; D,E), somatostatin (Sst; red; F,G), and PP (red; H,I). No significant colocalization of glucagon with other hormones was seen in control or IKO mice. Male control and IKO mice were used for analysis though female control and IKO mice produced similar results. Scale bar denotes 75 µm. (**J**): Endocrine cell number quantification for insulin, glucagon, somatostatin, and PP in control and IKO animals (over 10,000 cells were counted from 3 animals per group). (**K–L**): Quantitative PCR analysis of gene expression in control and IKO islets. Results are displayed as fold change relative to control with error bars representing the standard error of the mean. For all analysis n = 3.

To explore the requirement for *Arx* in the maintenance of adult α-cell fate, we used a global, tamoxifen-inducible transgenic mouse model to ablate *Arx* in adult animals. Two-month-old control and *Arx* ^L^/_Y_;*pCAGG-CreER* (IKO) mice were injected with tamoxifen for three consecutive days and the animals sacrificed two weeks later for tissue analysis (Fig. 5A). Efficiency of *Arx* removal was evaluated by immunostaining in control and IKO mice. While Arx expression was found in glucagon^+^ cells in control animals, all glucagon cells in IKO animals have lost Arx expression (Fig. 5B, B’, C, C’; marked by arrows). To determine the impact of short-term *Arx* ablation in adult α-cells, gene expression and immunostaining for endocrine hormones were examined in control and IKO animals (Fig. 5D–I). Real-time PCR analysis revealed no significant changes in the mRNA levels of hormone genes between control and IKO islets (Fig. 5K). We also did not detect any significant changes in the numbers of glucagon-, insulin-, somatostatin-, and PP-producing cells in the IKO mice compared to controls (Fig. 5D–J). Unlike P5 GKO mice in which a large proportion of Arx^-^glucagon^+^insulin^+^ cells were found (Fig. 2A–B), we detected only a small number of bihormonal cells in adult IKO mice, which were not proportionally significant (less than 0.1%; data not shown). Additionally, analysis of α- and β-cell factors including MafB, Brn4, Glut2, and Pdx1 also did not reveal any significant changes in transcriptional profile of IKO animals (Fig. 5L). Since the pCAGG-Cre is globally expressed, the IKO animals develop an intestinal phenotype that excludes any meaningful analysis to explore the long-term impact of *Arx* on adult α-cell (data not shown). Taken together, using our current mouse model with short-term Arx ablation, these findings demonstrate that *Arx* is likely dispensable in maintaining α-cell identity in adult mice. Future experiments utilizing an inducible α-cell specific Cre transgenic mouse will be required to study the long-term requirement for *Arx* in the maintenance of α-cell fate.

## Discussion

**Figure 6:**
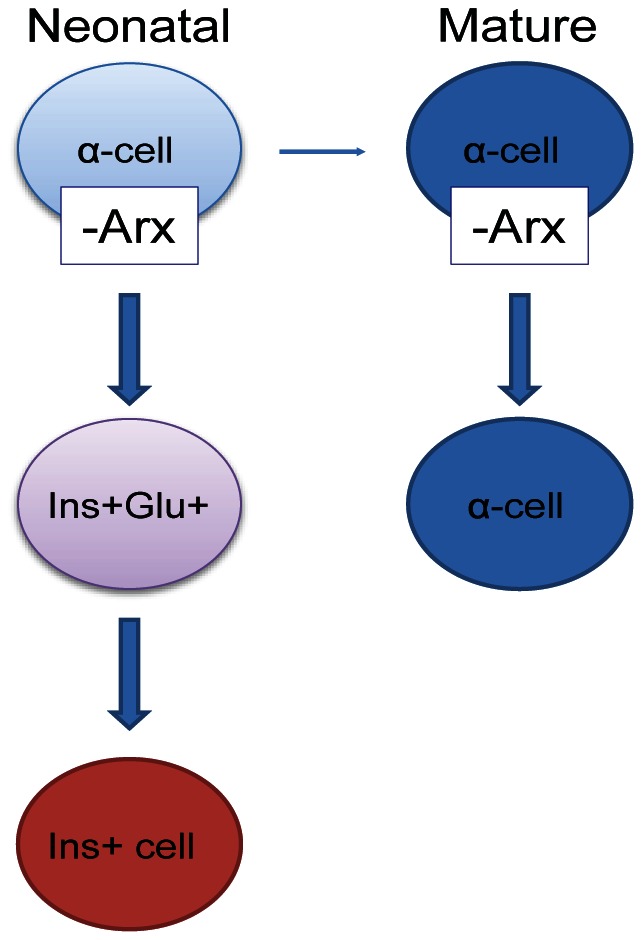
Proposed model showing that Arx is necessary to maintain α-cell fate in neonatal islets but not in mature α-cells in adult animals. Loss of neonatal Arx results in the conversion of glucagon^+^ α-cells into an insulin-producing β-like-cell through a bihormonal intermediate.

This study demonstrates a requirement of *Arx* in α-cell fate maintenance. Ablation of *Arx* in neonatal glucagon^+^ cells results in a loss of α-cell identity and conversion into an insulin-producing β-cell-like fate (Fig. 6). Conversely, short-term ablation of *Arx* in adult animals did not result in a significant loss or conversion of α-cells or an increase in β-cells or β-cell markers (Fig. 6). Our findings from neonates and adults expand the previously defined role of *Arx* in the specification of α-cells. When taken together, *Arx* plays a role during specification as well as during early maintenance of α-cell fate, but appears not to be required in adult animals for its fate maintenance.

Others have shown that ablation of *Arx* at any stage of specification results in a complete loss of the α-cell lineage with a concomitant increase of β- and δ-cells [9], [10], [12], [23]. As there is no change in total endocrine mass reported in these studies, these α-cells likely undergo re-specification into β- and δ-cell lineages. Our findings add significant support to these observations by using lineage tracing to directly demonstrate that *Arx*-ablated α-cells convert to β-like cells in neonatal animals. Interestingly, our data, while showing coexpression of glucagon and insulin, does not show coexpression of glucagon and somatostatin. Our initial hypothesis was that *Arx*-deficient α-cells would give rise to both somatostatin and insulin populations. It is possible that the inefficiency of the *Glucagon-Cre* did not enable us to detect a rare population of glucagon^+^somatostatin^+^ cells. Alternatively, as the animal ages, the plasticity of cells among different endocrine fates could be altered.

Previous studies have demonstrated that endocrine cell fate is relatively undifferentiated during gestation such that ablation of single transcription factors results in loss of cell fate [2]. As endocrine cells mature, however, this plasticity drastically decreases, and more extreme measures are needed to convert one endocrine cell type to another [4], [6], [8]. While *Pdx1* is normally restricted to β-cells, early overexpression in α-cells results in a postnatal loss of glucagon-expressing α-cells with a concomitant gain of insulin-producing β-cells demonstrating an α-to-β-cell fate conversion [8]. Conversely, overexpression of *Pdx1* in adult α-cells does not result in a similar conversion; instead, these cells maintain proper cell identity [8]. The potential temporal requirement of *Arx* closely parallels the results obtained through *Pdx1* overexpression in α-cells. Early in development, endocrine cell fate appears more plastic and subject to reprogramming. During later life, however, cell fate is more defined, and as a result, reprogramming is more difficult to achieve.

Interestingly, although overexpression of *Pdx1* in α-cells results in a gain of insulin-producing cells, those cells did not appear to lose all markers of α-cell fate [8]. Examination of immunostaining for the expression of β-cell-specific factors in the *Arx* ablated neonatal animals demonstrates that YFP^+^ cells are at least partially reprogrammed with the expression of Glut2, MafA, and Pdx1. While *Arx* is necessary to maintain α-cell fate during development, loss of *Arx*, even immediately after specification, may not be sufficient to fully reprogram cells into a functional β-cell fate. Due to the low efficiency of the *Cre* utilized in our study, functional analysis of the insulin-producing cells derived from *Arx* ablation in α-cells was not feasible.

As the animal ages, there could be epigenetic changes that have occurred during the process of specification or maturation that inhibit these *Arx* deficient cells from becoming functional β-cells under homeostatic conditions. In fact, epigenetic modification has been shown to play important roles in the differentiation and maintenance of cell types. A recent study demonstrates that α-to-β-cell reprogramming could be promoted by manipulating the histone methylation signature in mammalian pancreatic islets [24]. Conditions of stress, however, may also make cell fate transitions more fluid. It has been shown that excessive loss of β-cell mass, induced by administration of a β-cell specific toxin, results in spontaneous reprogramming of α-cells into a β-cell fate [6]. Additionally, partial pancreatectomy in mice and rats can result in regeneration of β-cells through the conversion of duct cells or duct progenitor cells [25], [26]. These studies demonstrate that while cell fate is more defined in adult animals, extreme conditions can force a non-β-cell into a β-cell fate. Future studies examining this possibility should be performed and will elucidate limits to cell fate maintenance in adult animals and how to overcome those limits. Particularly, the ability to utilize α-cells for conversion to functional β-cells could be a potential therapy for diabetes.

Finally, it is important to note that our current adult IKO mouse model does not allow for a complete investigation for the role of *Arx* in adult α-cells. *Arx* is required in early enteroendocrine cell development of the digestive tract [27]. Therefore mice with *Arx* removal in the intestine have alterations of specific enteroendocrine cell population, which lead to lipid malabsorption and diarrhea ([27]; and unpublished observations). Since the adult IKO mouse model was generated using a global inducible Cre transgenic mouse, enteroendocrine cell populations were impacted (unpublished observations). It is important to note that we did notice a 0.1% increase in the number of bihormonal cells in IKO mice (data not shown). However, whether this small change is due to the direct impact upon *Arx* loss in α-cells or changes in the animal’s physiology remains to be determined. An α-cell specific inducible *Arx-*deficient mouse model combined with lineage tracing studies will be required to precisely determine the role of *Arx* in adult α-cells.

In conclusion, the current study demonstrates a potential temporal requirement for *Arx* in maintenance of α-cell fate. Ablation of *Arx* in neonatal α-cells results in a loss of glucagon expression and a conversion of this cell population to adopt an insulin-producing β-cell-like fate. However, short-term loss of *Arx* in adult animals does not phenocopy this result but instead suggests that *Arx* is dispensable in maintaining α-cell fate in adulthood. These data expand the knowledge of the field not only related to the role of *Arx* in the endocrine α-cell but also in regards to global temporal restrictions for reprogramming endocrine cells. Future studies examining this temporal requirement, as well as perturbations to the cell that circumvent these restrictions, will help clarify this plasticity and bring understanding to endocrine cell fate specification, maintenance, and therapeutic potential.

## Supporting Information

Figure S1 Arx is specifically ablated in P21 α-cells by *Glucagon-Cre* with *Arx-*deficient cells expressed YFP. Pancreata were stained for glucagon (blue), Arx (red), and YFP (green). **(A):** Arx is expressed in all glucagon^+^ cells in control;*Rosa-YFP* pancreata. **(B):** In GKO;Rosa-YFP animals, Arx is ablated in all YFP^+^ cells. Male and female GKO mice (n≥3) were analyzed and compared to their sex-matched controls. Scale bar represents 50 µm.
(TIF)

Figure S2 Loss of glucagon^+^insulin^+^ cells in P21 GKO animals. **(A–H):** Immunostaining for glucagon (A–H), insulin (A–B), somatostatin (C–D), PP (E–F), and ghrelin (G–H) in P21 control and GKO animals with merged images shown. Glucagon positive cells do not overlap with insulin (A, B), somatostatin, (C, D) or PP (E, F). Ghrelin is no longer expressed at P21 (G, H). **(I):** Total insulin, glucagon, somatostatin, and PP cell mass in the pancreata of P21 control and GKO mice. Male and female GKO mice (n≥3) were analyzed and compared to their sex-matched controls. Scale bar denotes 25 µm. **(J):** Quantitative PCR analysis for P21 control and GKO islets for β-cell markers Pdx1 and Nkx6.1 and α-cell markers MafB and Brn4. “*” denotes p<0.05. Error bars represents standard error of the mean.
(TIF)

Figure S3 YFP^+^ cells in GKO animals express markers of mature β-cells at P21. **(A–F):** Control;Rosa-YFP and GKO;Rosa-YFP P21 pancreata stained for insulin (blue), YFP (green), Glut2 (A,B,red), MafA (C,D,red), and Pdx1 (E,F,brown). YFP^+^ cells in GKO animals are insulin^+^Glut2^+^ (B), insulin^+^MafA^+^ (D) and insulin^+^Pdx1^+^ (F) and are not found in control animals (A,C,E). YFP staining in the exocrine tissue in E and F is non-specific and is a result of combined IHC/IF protocol. (←) in all panels denotes YFP cells expressing various β-cell markers. Male and female GKO mice (n≥3) were analyzed and compared to their sex-matched controls. Scale bar represents 25 µm.
(TIF)
