# Supplementary figures and images for: Pancreatic α-Cell Specific Deletion of Mouse Arx Leads to α-Cell Identity Loss
Source: PLoS One. 2013 Jun 13;8(6):e66214. doi: 10.1371/journal.pone.0066214 (PMC3681972; doi:10.1371/journal.pone.0066214)

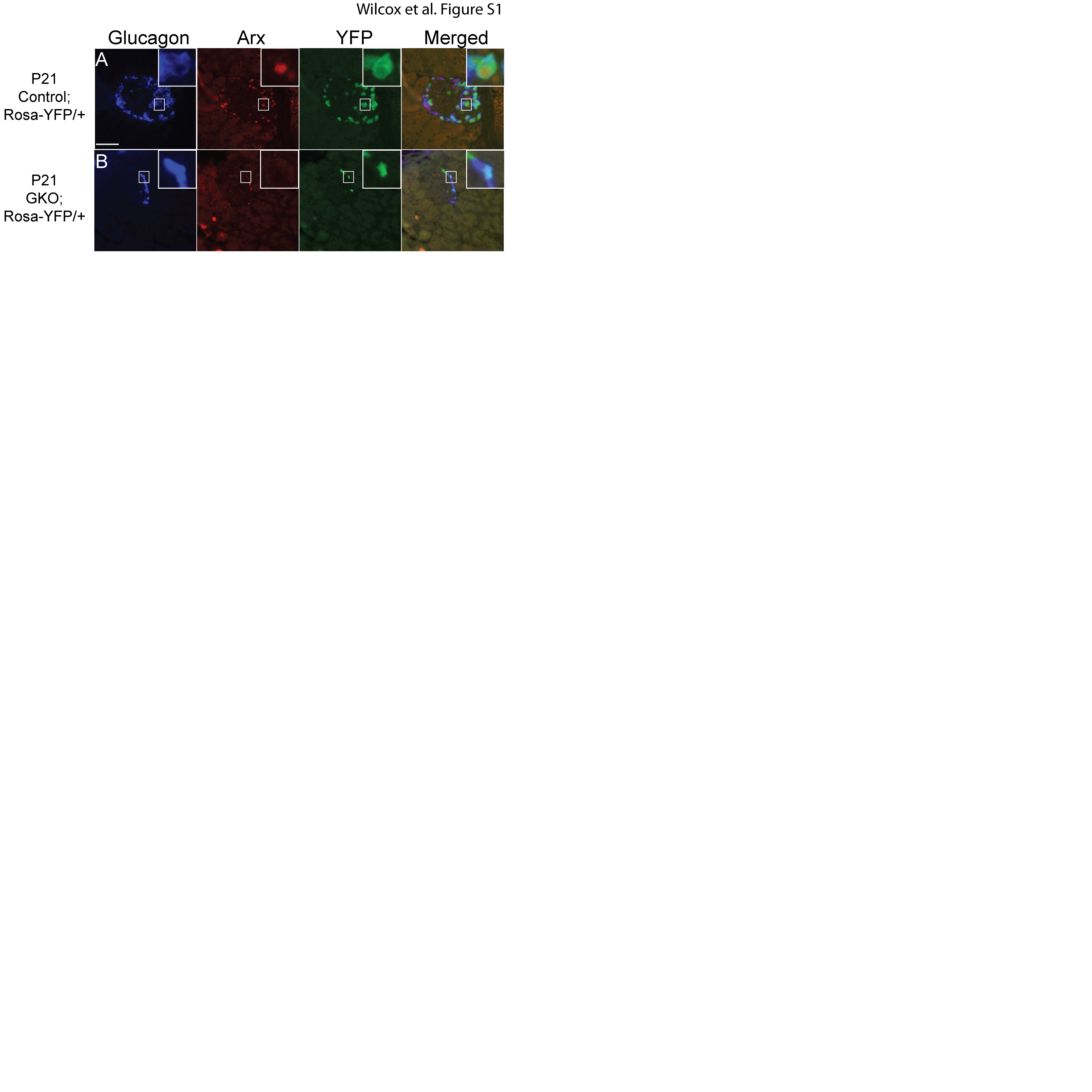

Supplement: Figure S1 — Arx is specifically ablated in P21 α-cells by Glucagon-Cre with Arx-deficient cells expressed YFP. Pancreata were stained for glucagon (blue), Arx (red), and YFP (green). (A): Arx is expressed in all glucagon+ cells in control;Rosa-YFP pancreata. (B): In GKO;Rosa-YFP animals, Arx is ablated in all YFP+ cells. Male and female GKO mice (n≥3) were analyzed and compared to their sex-matched controls. Scale bar represents 50 µm. (TIF) [file pone.0066214.s001.tif]

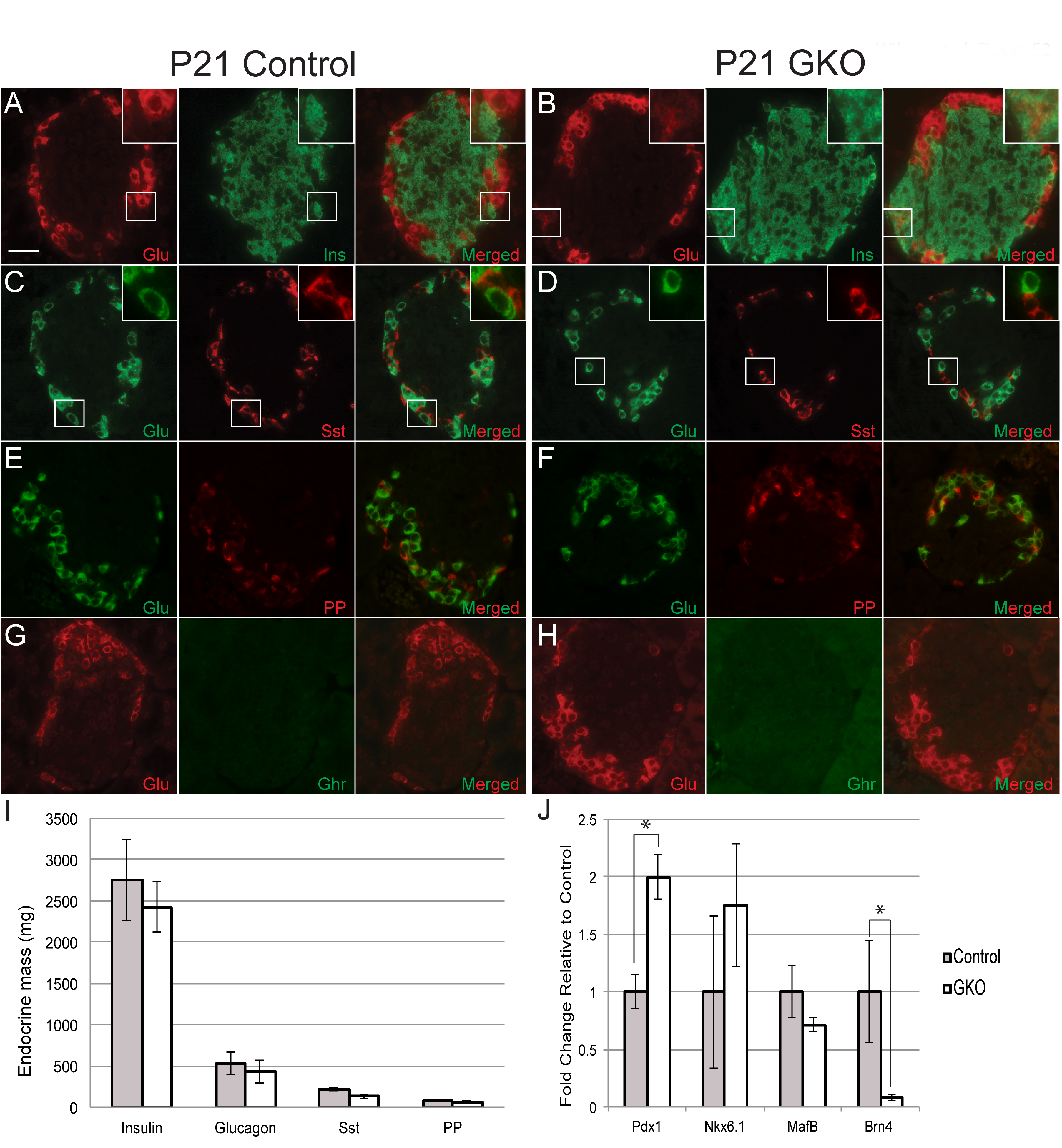

Supplement: Figure S2 — Loss of glucagon+insulin+ cells in P21 GKO animals. (A–H): Immunostaining for glucagon (A–H), insulin (A–B), somatostatin (C–D), PP (E–F), and ghrelin (G–H) in P21 control and GKO animals with merged images shown. Glucagon positive cells do not overlap with insulin (A, B), somatostatin, (C, D) or PP (E, F). Ghrelin is no longer expressed at P21 (G, H). (I): Total insulin, glucagon, somatostatin, and PP cell mass in the pancreata of P21 control and GKO mice. Male and female GKO mice (n≥3) were analyzed and compared to their sex-matched controls. Scale bar denotes 25 µm. (J): Quantitative PCR analysis for P21 control and GKO islets for β-cell markers Pdx1 and Nkx6.1 and α-cell markers MafB and Brn4. “*” denotes p<0.05. Error bars represents standard error of the mean. (TIF) [file pone.0066214.s002.tif]

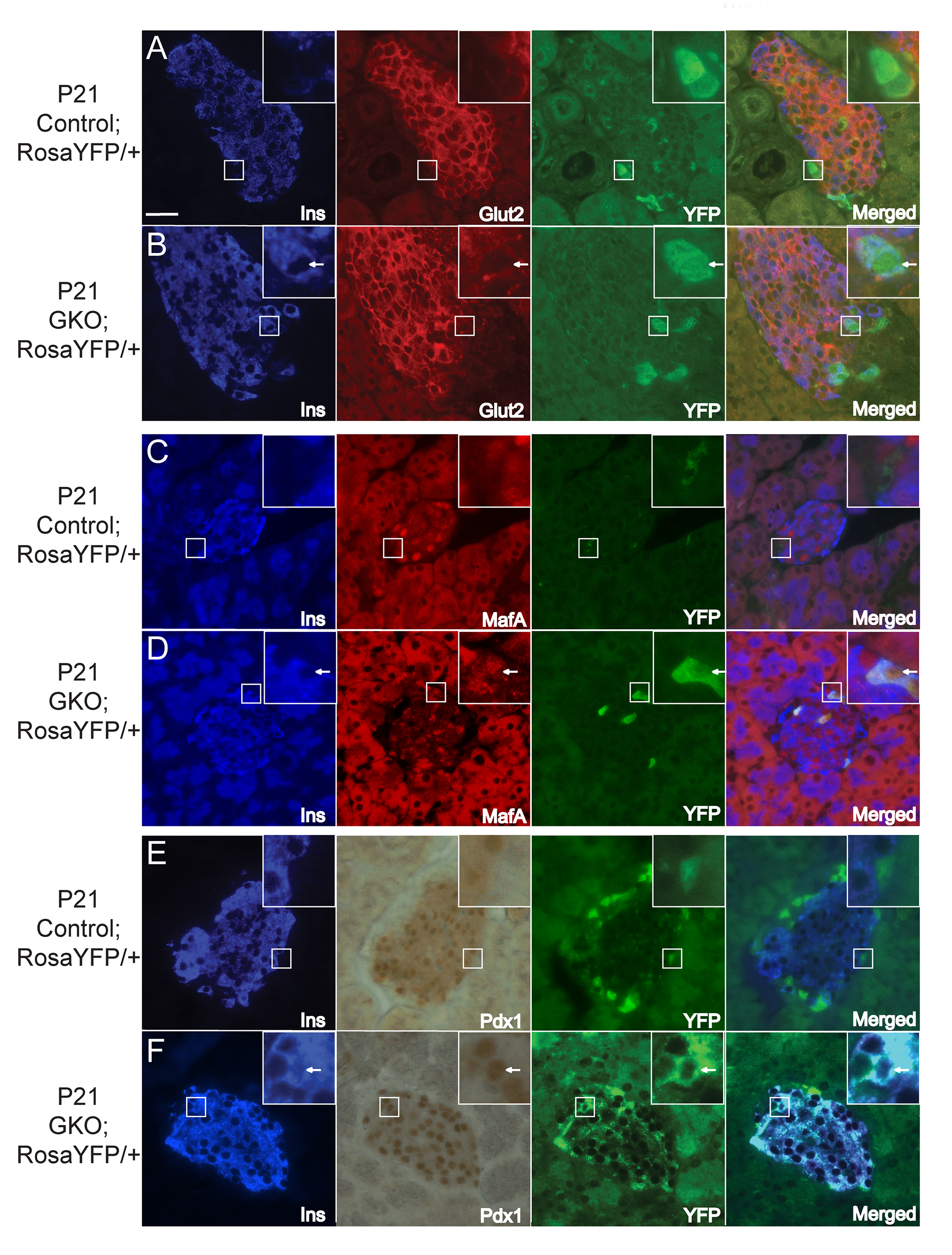

Supplement: Figure S3 — YFP+ cells in GKO animals express markers of mature β-cells at P21. (A–F): Control;Rosa-YFP and GKO;Rosa-YFP P21 pancreata stained for insulin (blue), YFP (green), Glut2 (A,B,red), MafA (C,D,red), and Pdx1 (E,F,brown). YFP+ cells in GKO animals are insulin+Glut2+ (B), insulin+MafA+ (D) and insulin+Pdx1+ (F) and are not found in control animals (A,C,E). YFP staining in the exocrine tissue in E and F is non-specific and is a result of combined IHC/IF protocol. (←) in all panels denotes YFP cells expressing various β-cell markers. Male and female GKO mice (n≥3) were analyzed and compared to their sex-matched controls. Scale bar represents 25 µm. (TIF) [file pone.0066214.s003.tif]
